# The effects of vocal exertion on lung volume measurements and acoustics in speakers reporting high and low vocal fatigue

**DOI:** 10.1371/journal.pone.0268324

**Published:** 2022-05-12

**Authors:** Robert Brinton Fujiki, Jessica E. Huber, M. Preeti Sivasankar

**Affiliations:** 1 Department of Surgery, University of Wisconsin-Madison, Madison, WI, United States of America; 2 Department of Speech, Language, and Hearing Sciences, Purdue University, West Lafayette, IN, United States of America; University of Colorado School of Medicine, UNITED STATES

## Abstract

**Purpose:**

Vocal exertion is common and often results in reduced respiratory and laryngeal efficiency. It is unknown, however, whether the respiratory kinematic and acoustic adjustments employed during vocal exertion differ between speakers reporting vocal fatigue and those who do not. This study compared respiratory kinematics and acoustic measures in individuals reporting low and high levels of vocal fatigue during a vocal exertion task.

**Methods:**

Individuals reporting low (N = 20) and high (N = 10) vocal fatigue participated in a repeated measures design study over 2 days. On each day, participants completed a 10-minute vocal exertion task consisting of repeated, loud vowel productions at elevated F0 sustained for maximum phonation time. Respiratory kinematic and acoustic measures were analyzed on the 1^st^ vowel production (T0), and the vowels produced 2 minutes (T2), 5 minutes (T5), 7 minutes (T7), and 10 minutes (T10) into the vocal exertion task. Vowel durations were also measured at each time point.

**Results:**

No differences in respiratory kinematics were observed between low and high vocal fatigue groups at T0. As the vocal exertion task progressed (T2-T10), individuals reporting high vocal fatigue initiated phonation at lower lung volumes while individuals with low vocal fatigue initiated phonation at higher lung volumes. As the exertion task progressed, total lung volume excursion decreased in both groups. Differences in acoustic measures were observed, as individuals reporting high vocal fatigue produced softer, shorter vowels from T0 through T10.

**Conclusions:**

Individuals reporting high vocal fatigue employed less efficient respiratory strategies during periods of increased vocal demand when compared with individuals reporting low vocal fatigue. Individuals reporting high vocal fatigue had shorter maximum phonation time on loud vowels. Further study should examine the potential screening value of loud maximum phonation time, as well as the clinical implications of the observed respiratory patterns for managing vocal fatigue.

## Introduction

Laryngeal pathology hinders communication for more than 17 million adults in the United States [1]. Although the precise etiology of vocal pathology is varied, some voice disorders may result from the accrued loss of laryngeal and respiratory efficiency caused by vocal exertion (also referred to as vocal loading in the laboratory) [2, 3]. Vocal exertion is common in both social and professional situations [4–8], and can negatively impact vocal fold physiology, as well as make phonation more difficult [9, 10]. For example, vocal exertion increases self-perceived vocal effort [11–13], and often induces changes in acoustic voice measures [14–16], such as increased fundamental frequency or truncated vocal range [17–19]. Vocal exertion may also adversely affect voice quality as indicated by changes in cepstral measures or measures of perturbation [13, 14, 20]. In addition, vocal exertion may increase viscoelastic vocal fold properties as demonstrated by changes in aerodynamic measures [21–23].

Vocal exertion may also affect respiratory function since respiratory kinematics drive vocal intensity and are correlated with vocal effort [24–28]. Recent research has considered changes in respiratory kinematics resulting from vocal exertion [17, 29, 30]. For example, healthy older adults initiated speech at lower lung volumes following a loud reading task, suggesting possible fatigue [30]. These changes were not evident, however, in young healthy adults or teachers following loud reading tasks [29, 30]. Young healthy adults initiated speech at higher lung volumes following a sustained-vowel task, presumably to benefit from recoil forces and increase respiratory efficiency in the face of vocal challenge [17]. This might be expected considering that initiating speech at higher lung volumes can be an efficient mechanism to increase vocal intensity [24, 31, 32]. Additional research comparing populations across identical vocal exertion tasks is needed to better understand how respiratory kinematics are affected by vocal exertion.

Although changes in vocal effort, voice measures, and respiratory kinematics are frequently evident following vocal exertion [29, 33–36], the duration and magnitude of these changes vary [37, 38]. In laboratory conditions, changes in aerodynamic and acoustic voice measures have lasted for as little as ten minutes or as long as two hours [17, 21, 39]. These differences in outcomes are likely tied to the extent or type of vocal exertion experienced [40]. Even in studies using exertion tasks that are relatively similar, the results of vocal exertion may vary across individuals [40, 41] and repeated days [20, 42]. In addition, increases in vocal effort may occur and persist in the absence of negative changes to objective voice measures [43]. Additionally, these changes may last even longer in individuals who experience voice problems such as vocal fatigue.

Emerging evidence suggests that individuals who experience vocal fatigue respond differently following periods of high vocal demand than their vocally healthy counterparts [34, 44, 45]. This is perhaps not surprising as vocal fatigue is characterized by detrimental self-perceptual or physiological voice changes induced by extended voice use [46–49]. Differences in vocal demand response in people with high vocal fatigue have been observed primarily in respiratory kinematics, which have been shown to differ in individuals with structural and functional voice disorders [3, 50, 51]. For example, following a loud, prolonged vowel task, individuals reporting high vocal fatigue initiated speech at lower lung volumes [34]. Following the same task, individuals with low vocal fatigue increased lung volume initiation, presumably to benefit from increased recoil forces and thereby improve respiratory efficiency [17]. Similarly, teachers experiencing vocal fatigue were observed to initiate and terminate speech at lower lung volumes than their vocally healthy counterparts during teaching tasks [3]. As such, it appears that there is a relationship between vocal fatigue and respiratory kinematics following vocal exertion [44].

Although individuals reporting chronic vocal fatigue may use different respiratory strategies following vocal exertion when compared with vocally healthy individuals, it is unclear exactly when speakers implement these strategies. Do individuals make physiological compensations during the task, or are these changes only evident following vocal exertion? Voice and respiratory kinematic measures are usually collected before and after vocal exertion rather than during the vocally exerting events themselves [19, 52–55]. Therefore, little is known regarding the compensatory strategies speakers use to successfully complete vocally demanding tasks in the moment. It is also unknown if these strategies change over multiple trials of a vocal exertion task. Investigating the way individuals reporting high and low levels of vocal fatigue respond to vocal exertion has the potential to increase our understanding of how vocal exertion and vocal fatigue intersect. It is hypothesized that exertion induced changes in laryngeal function, changes in respiratory kinematics, and vocal fatigue are all inter-related and possible cyclical (S1 Fig). It is unclear, however, whether loss of laryngeal efficiency, changes in respiratory kinematics, or vocal fatigue initiate this cycle. Understanding what changes occur during actual moments of vocal exertion will inform our understanding of how vocal fatigue should best be managed clinically.

This study examined respiratory kinematics and acoustic measures in individuals reporting low and high levels of vocal fatigue when completing a vocal exertion task across 2 experimental days. The vocal exertion task consisted of repeated, loud, sustained vowels. The vowels were sustained for maximum duration on a single breath, at 50^th^ of frequency range. It was hypothesized that individuals reporting high levels of vocal fatigue in daily life would utilize lower lung volumes to complete the vocal exertion task than their counterparts with low vocal fatigue. It was hypothesized that acoustic voice measures would change as the exertion task progressed, and that these changes would be more adverse for the high vocal fatigue group than the low fatigue group. It was also hypothesized that the detrimental respiratory and acoustic changes would be of greater magnitude on day 2.

## Methods

### Study procedures

All study procedures were approved by the Purdue University Institutional Review Board (IRB# 1805020623). These data were gathered as part of a larger dissertation study, and the laryngeal and respiratory data collected prior to and following this vocal exertion task have been previously published [17, 34]. This current study examined laryngeal and respiratory measures during the vocal exertion task itself.

**Fig 1:**
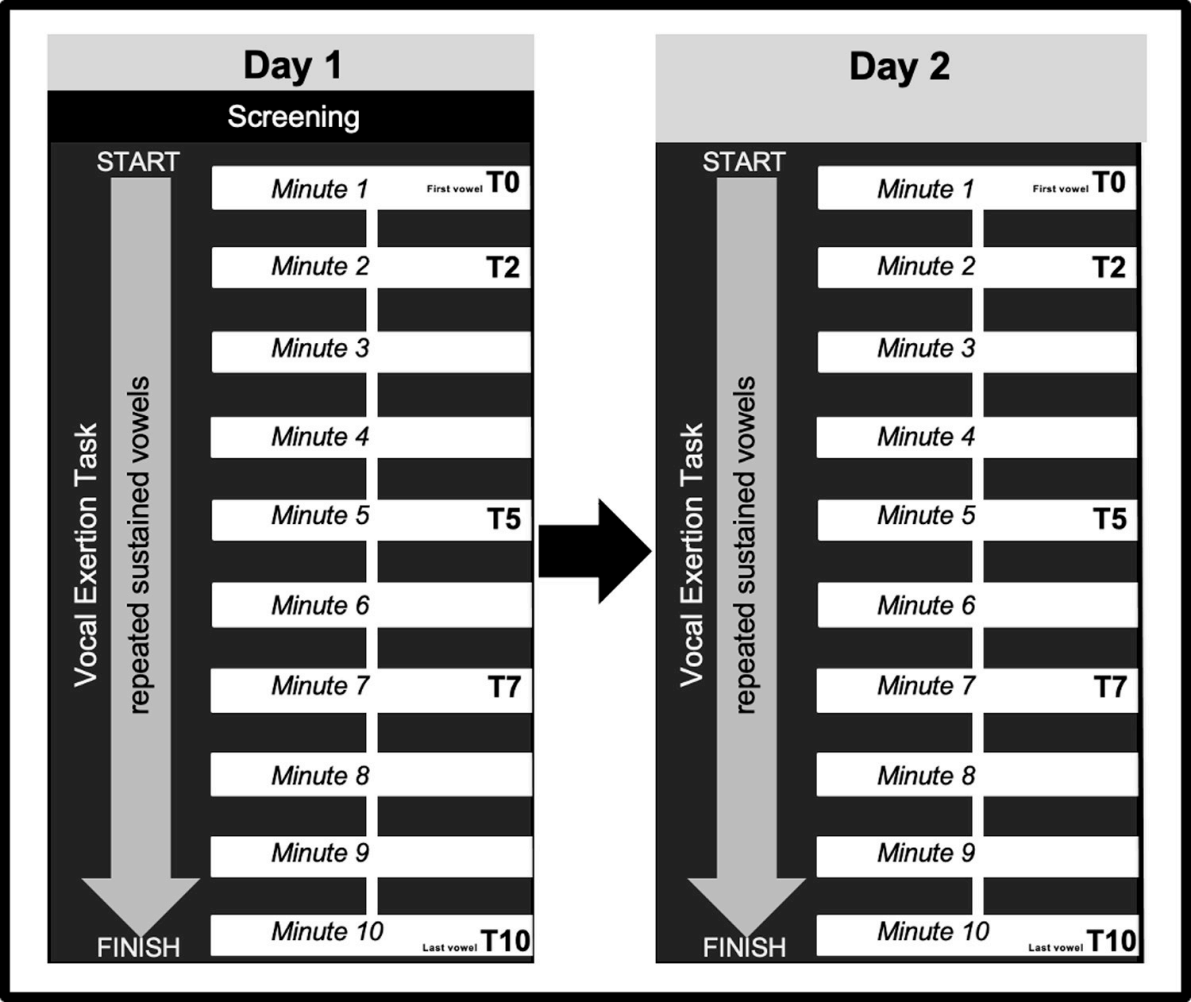
Visual representation of study design.

A repeated measures design was utilized. Participants carried out two identical vocal exertion tasks on consecutive days. Vocal exertion tasks consisted of ten minutes of repeated loud, sustained vowels at elevated fundamental frequency (F0) [17, 56]. Experimental days were scheduled 24 hours apart (+ or– 1 hour) and were identical for the portions of the protocol examined in this study, except that consent and screening procedures were performed on day one. Participants followed similar patterns of voice use and food/liquid intake for 24 hours prior to each experimental day. During each experimental day, the vocal exertion task consisted of multiple loud, sustained vowels at elevated fundamental frequency (F0) [17, 56]. These vowel productions were repeated until 10 minutes had elapsed. Respiratory kinematic and acoustic data were collected throughout the vocal exertion task (described below) on both days. All participants completed the full vocal exertion task on both experimental days, however, five sustained vowels from each vocal exertion task were selected for analysis: the first vowel production (T0) and the vowels produced closest to the following time markers; 2 minutes (T2), 5 minutes (T5), 7 minutes (T7), and 10 minutes (T10) into the vocal exertion task. The vowel produced at the 10-minute marker (T10) was the final prolonged vowel of the vocal exertion task. The study design is presented in Fig 1.

### Participants

Participants were recruited from the Purdue campus using flyers. Thirty participants passed screening procedures and were included in this study. Inclusionary criteria included (a) age greater or equal to 18 years, (b) general good health, and (c) perceptually normal vocal quality. Exclusionary criteria included (a) history of voice disorder, respiratory disease, hearing loss, or head/neck cancer, (b) history of voice training, or (c) self-reported history of smoking. Spirometry was used to screen participants (Discovery Spirometer, Futuremed America, Inc., Granada Hills, CA) for normal vital capacity and forced vital capacity (≥80% of expected values based on sex, age, ethnicity, weight, and height matched norms). In addition, rigid videostroboscopy (9100 KayPENTAX, Lincoln Park, NJ) was used to ensure that participants were free of any gross laryngeal pathology.

Participants were recruited into low and high vocal fatigue groups using the Vocal Fatigue Index (VFI) [57]. Prior to data collection, all participants completed the VFI, a validated, self-report questionnaire designed to quantify vocal fatigue. Participants who scored within normal range (<24 on part 1 and <7 on part 2) were assigned to the low vocal fatigue group, and participants scoring in the range indicative of vocal fatigue (≥24 on part 1 and/or ≥7 on part 2) were assigned to the high vocal fatigue group [57]. No participants in either group were experiencing vocal symptoms at the time of data collection.

Twenty individuals were categorized into the low vocal fatigue group (mean age = 20 years, SD = 3.08); 10 females, 10 males) and 10 individuals were categorized into the high vocal fatigue group (mean age = 20 years, SD = 3.03; 4 females, 6 males).

### Vocal exertion task

The 10-minute vocal exertion task consisted of repeated loud, sustained vowels at elevated F0 [17, 56]. Target vowel duration, intensity, and pitch were established prior to data collection during a practice production of the exertion task on day one. Participants were instructed to sustain each vowel production for maximum phonation time. Vowels were sustained at 50% pitch range as this pitch was within modal register but was elevated compared with comfortable speaking F0. Vocal range was calculated prior to data collection using pitch glides to maximal and minimum pitches of vocal range. Target vocal intensity was required to be greater than 80 dB (no upper limit) as measured by a sound level meter (24 inches from participant). Participants were also alerted if vowel duration or intensity fell below target levels. Participants were cued for target pitch using a digital keyboard prior to each production (Casio SA-76, Casio Computer Co. Ltd, Tokyo, Japan). Participants were notified when they were 5 minutes into the task, when 2 minutes remained, and when 1 minute remained. All participants in the low vocal fatigue group completed the task as described. Two participants in the high vocal fatigue group reported vocal fatigue and discomfort around the 5-minute marker (T5). In these cases, target pitch was reduced by a third to enable completion of the task.

### Outcome measures

#### Respiratory kinematics

**Fig 2:**
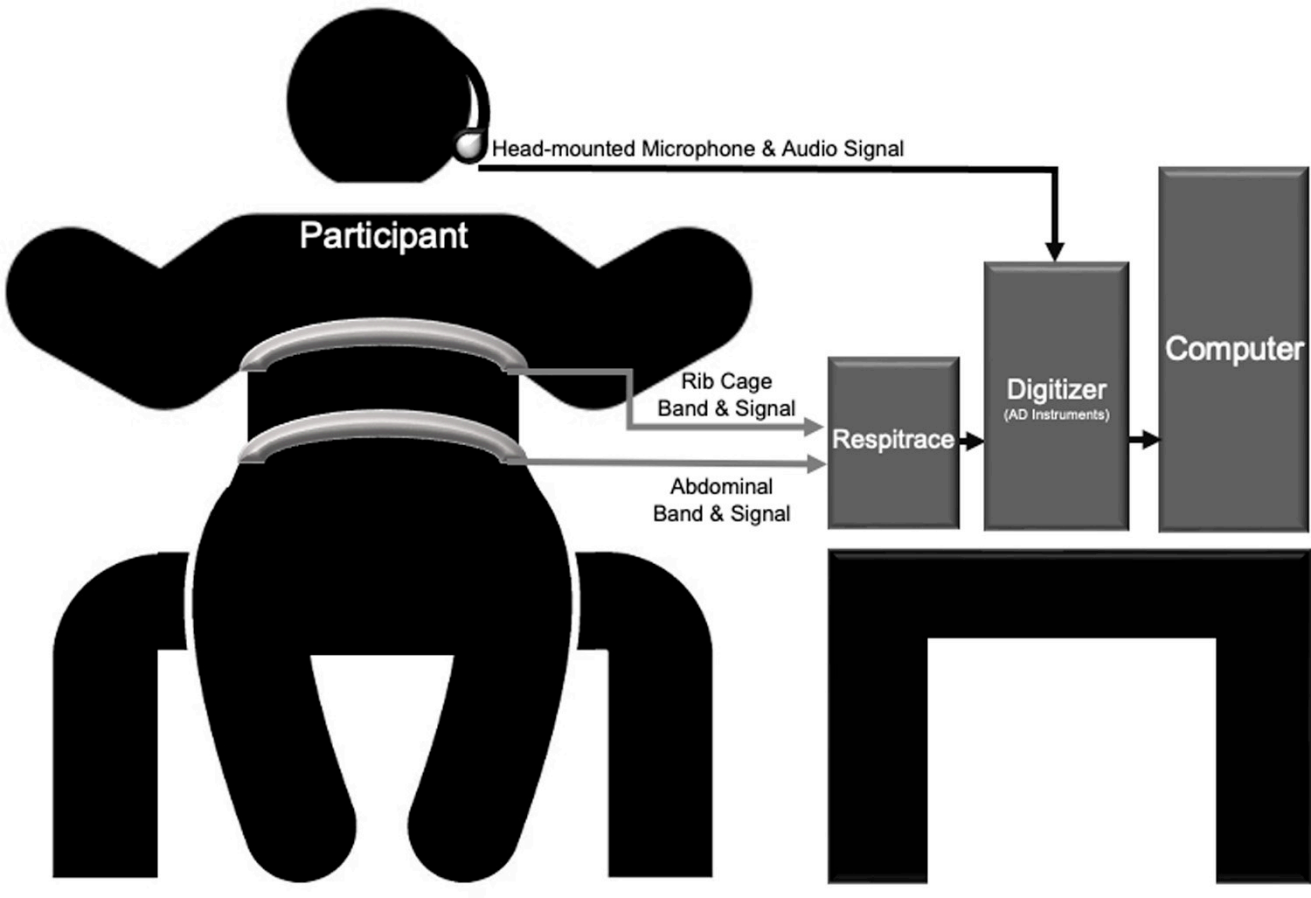
Schematic of equipment set-up.

Respiratory measures included lung volume initiation, termination, and excursion. Lung volumes were estimated using inductive plethysmography (Respitrace system, Ambulatory Monitoring, Inc., Ardsley, NY). Rib cage and abdomen movements were measured using respiratory bands placed on the patient’s rib cage (inferior to the axilla) and abdomen (inferior to rib 12) respectively. Respiratory bands were attached to participant clothing with tape. After respiratory bands were placed, participants remained seated for all data collection. Labchart was used to capture respitrace and audio signals synchronously. Respitrace system voltage was recorded into Labchart at a 1 kHz/s sampling rate. Equipment setup is presented in Fig 2.

Lung volumes were estimated from the corrected sum of rib cage and abdominal movements [58]. Correction factors were determined from periods of rest breathing and “speech-like” breathing (silently reading the phrase “You buy Bobby a puppy now if he wants one” during exhalation) [24, 25, 31, 32]. Participants breathed into a spirometer mouthpiece using a tight lip seal and wore nose clips during calibration procedures to ensure all air volume was directed through the spirometer mouthpiece. Rib cage and abdomen movements, as well as digital spirometer (FE141 ADInstruments, Colorado Springs, CO) measurements of lung volume, were recorded during rest and speech-like breathing periods. Digital spirometer volume output was compared with the sum of rib cage and abdomen movements during these calibration tasks [24, 25, 29, 59, 60]. Calibration coefficients (k_1_ and k_2_) for the rib cage and abdomen signals were calculated using a least squares solution (Matlab pseudoinverse function) with the formula

$$spirometer=k_{1}(rib\;cage)+k_{2}(abdomen).$$ (1)

The least squares calibration method has used respiratory kinematic data to estimate lung volumes within 5% accuracy of actual lung volume [59, 61]. During phonation, lung volumes were estimated using these calibration coefficients in the formula

$$Estimated\;Lung\;Volume=k_{1}(rib\;cage)+k_{2}(abdomen).$$ (2)

A vital capacity maneuver was also performed 3 times with the digital spirometer. This consisted of maximum inhalation and maximum exhalation.

Respiratory data were gathered during the vocal exertion task and were analyzed with an algorithm run using MATLAB (MathWorks, Inc., Natick, MA). To enable across-participant comparisons, respiratory data were calculated as a percent of vital capacity. Prior to the start of the vocal exertion task, a minimum of three troughs of rest breathing were collected to calculate end expiratory level (EEL).

- Lung volume initiation (LVI) was the lung volume at which phonation was initiated, and lung volume termination (LVT) was the lung volume at which phonation was terminated. Both were expressed as a percentage of vital capacity relative to EEL. Negative values reflect lung volumes below EEL and positive values reflect lung volumes above EEL.
- Lung volume excursion was the total lung volume displaced during phonation and represented the difference between LVI and LVT in percent vital capacity.

#### Acoustic measures

Acoustic measures included vowel duration, sound pressure level (SPL), fundamental frequency (F0), and cepstral peak prominence (CPP). A head-mounted microphone (AKG C555 L, AKG, Vienna, Austria; 2-inch mouth to microphone distance) was utilized for all audio-recordings. Audio signals were routed through an A/D converter (PowerLab 16/30 ADInstruments, Colorado Springs, CO) to a computer. All audio recordings were made at a 44kHz sampling rate. To allow for calculation of SPL, a 90 dB calibration tone (Acoustic calibrator Model QC-10/QC-20, Quest Technologies, Oconomowoc, WI) was recorded using the same equipment and gain level used to record data. Except for vowel duration, all acoustic analyses were performed on the middle 5 seconds of prolonged vowels.

- Duration of prolonged vowels in seconds was calculated using Praat (version 6.1.16, Phonetic Sciences, Amsterdam).
- Mean Sound Pressure Level (SPL) of prolonged vowels was calculated using Praat. The calibration tone at set gain was accounted for in the calculation of SPL.
- Fundamental frequency (F0) was calculated using Praat.
- Cepstral peak prominence (CPP) was analyzed using the sustained vowel protocol in the Analysis of Dysphonia in Speech and Voice software (ADSV Model 5109, KayPENTAX, Montvale, NJ).

#### Statistical analysis

Interclass correlation coefficients (ICCs) were calculated to measure inter-rater reliability using SPSS (Version 23, 2016). Reliability was calculated for 10% of respiratory kinematic and acoustic data.

The effects of the vocal exertion task across experimental days and groups were analyzed using mixed level modeling. Fixed factors in the model included time (T0, T2, T5, T7 and T10, within group), day (day one and two, within group), and group (low and high vocal fatigue groups, between group). All interactions were also included in the statistical model. All assumptions for mixed level modeling were met. Fisher’s protected least significant difference (LSD) tests were used for post-hoc comparisons for significant main effects and all significant interaction effects. Alpha level for significance was set at *p* < .05. Mixed models were run using SAS (Version 9.4, 2013).

## Results

### Reliability analysis

Inter-rater reliability for lung volume initiation and termination were in good range (LVI ICC = .88, *p* = .00, 95% [.68, .96]; LVT ICC = .87, *p* = .00, 95% [.67, .96]). Inter-rater reliability was in excellent range for vowel duration (ICC = .97, *p* = 00, 95% [.90, .99]), SPL (ICC = .90, *p* = 00, 95% [.75, .90]), F0 (ICC = .90, *p* = .00, 95% [.74, .96]), and CPP analyses (ICC = .96, *p* = 00, 95% [.88, .98]).

### Respiratory measures

#### Lung volume initiation (LVI)

**Fig 3:**
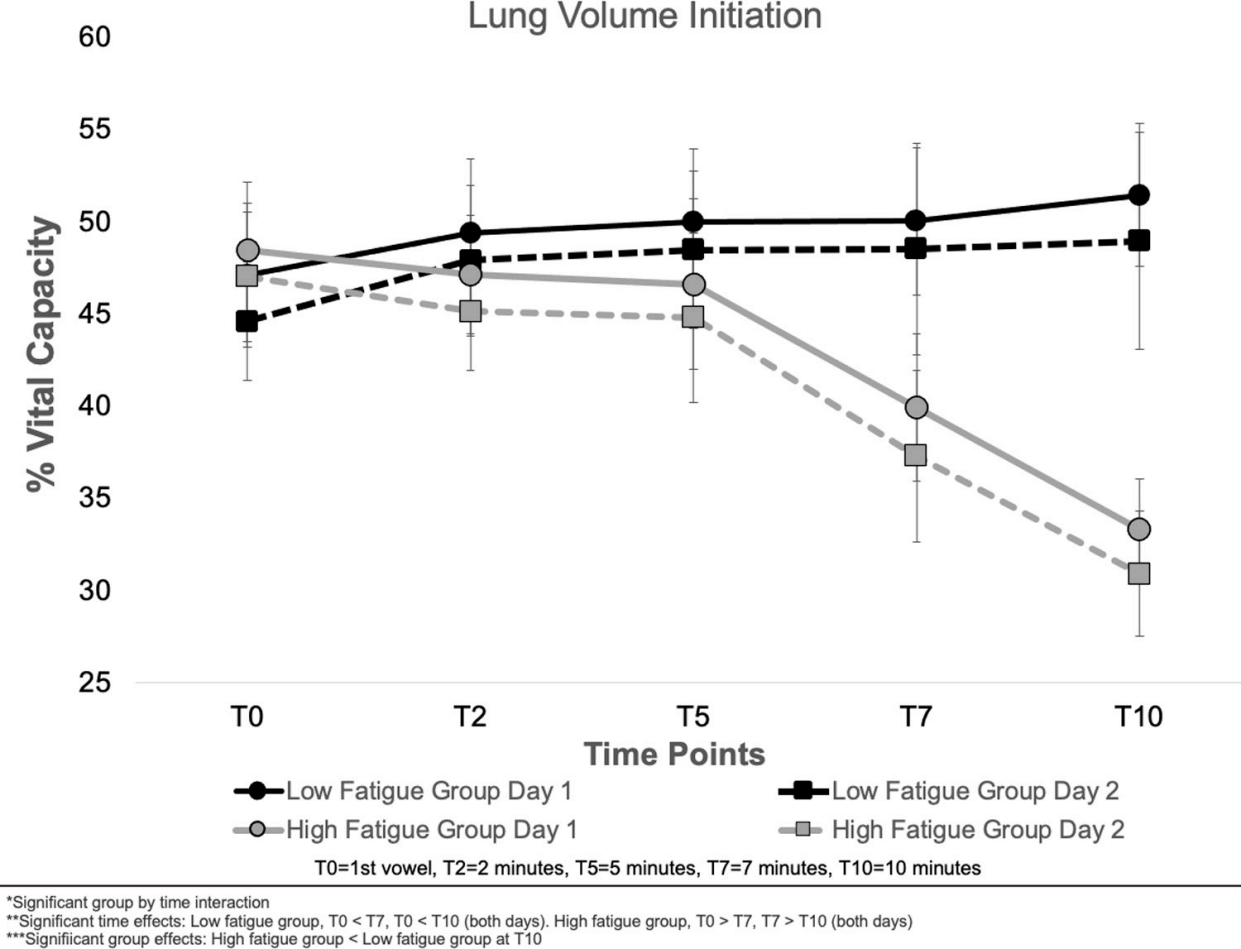
Means and standard errors for lung volume initiation across all time points, experimental days, and low and high vocal fatigue groups.

Means and standard errors for LVI are presented in Fig 3. A significant group by time interaction was also observed for LVI (F (4, 112) = 8.80, *p* < .001). For the low vocal fatigue group, LVI was significantly higher at T7 (*p* = .039) and T10 (*p* = .007), when compared with T0 on both days. For the high vocal fatigue group, LVI was significantly lower than T0 at T7 (*p* = .013) and significantly lower than T7 at T10 (*p* < .001). LVI data did not significantly differ between the low and high vocal fatigue groups except at T10 when data were significantly lower for the high vocal fatigue group (*p* = .008). No significant effect of day or interaction between day, time, and group was observed.

#### Lung volume termination (LVT)

**Fig 4:**
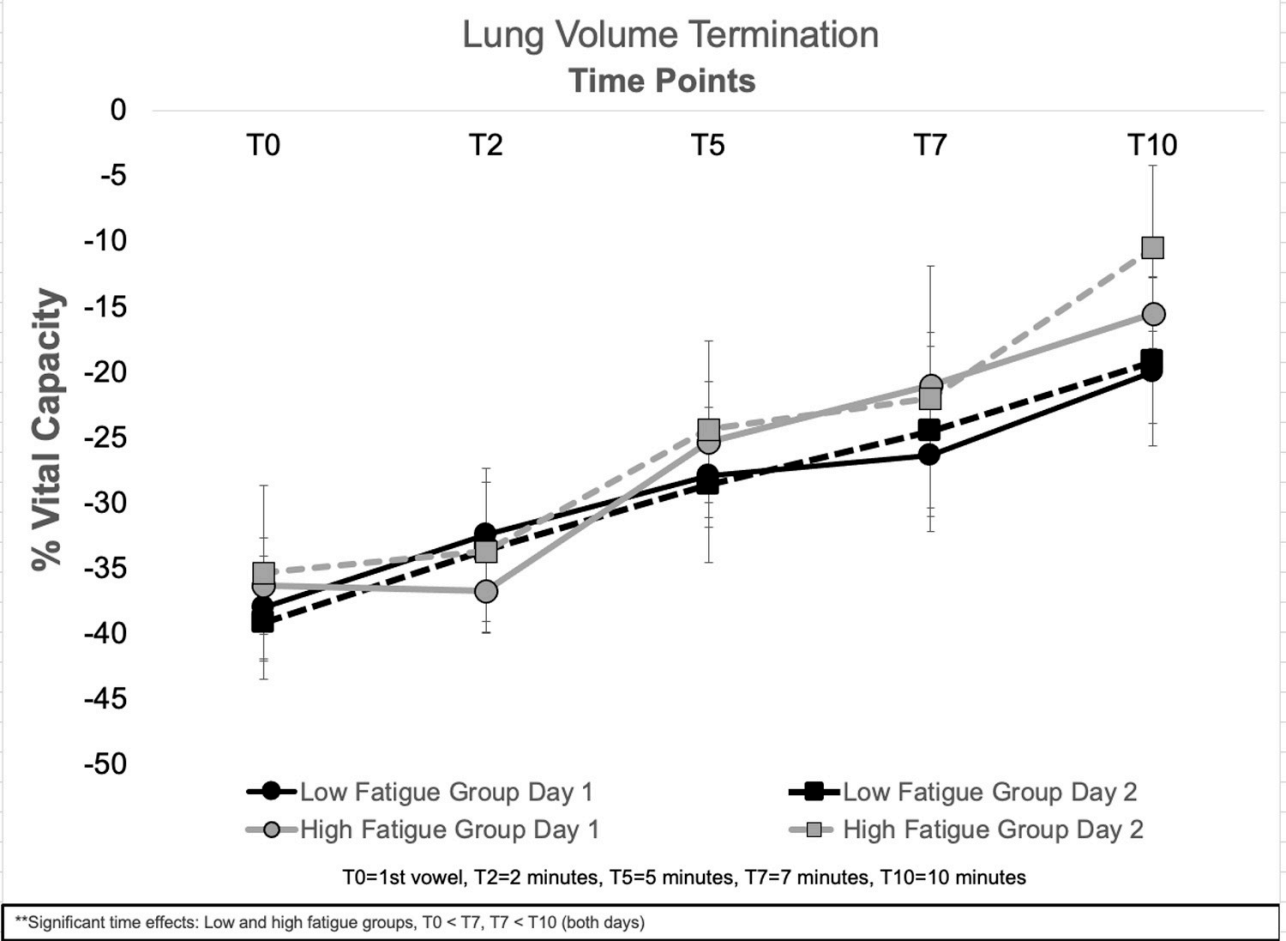
Means and standard errors for lung volume termination across all time points, experimental days, and low and high vocal fatigue groups.

Means and standard errors for LVT are presented in Fig 4. A significant main effect of time was observed for LVT (F (4, 112) = 17.65, *p* < .001). For both groups, LVT was significantly higher at T7 than T0 (*p* < .001); and significantly higher at T10 than T7 (*p* = .018). No significant main effects of group, day, or significant interactions were observed for LVT.

#### Lung volume excursion (LVE)

**Fig 5:**
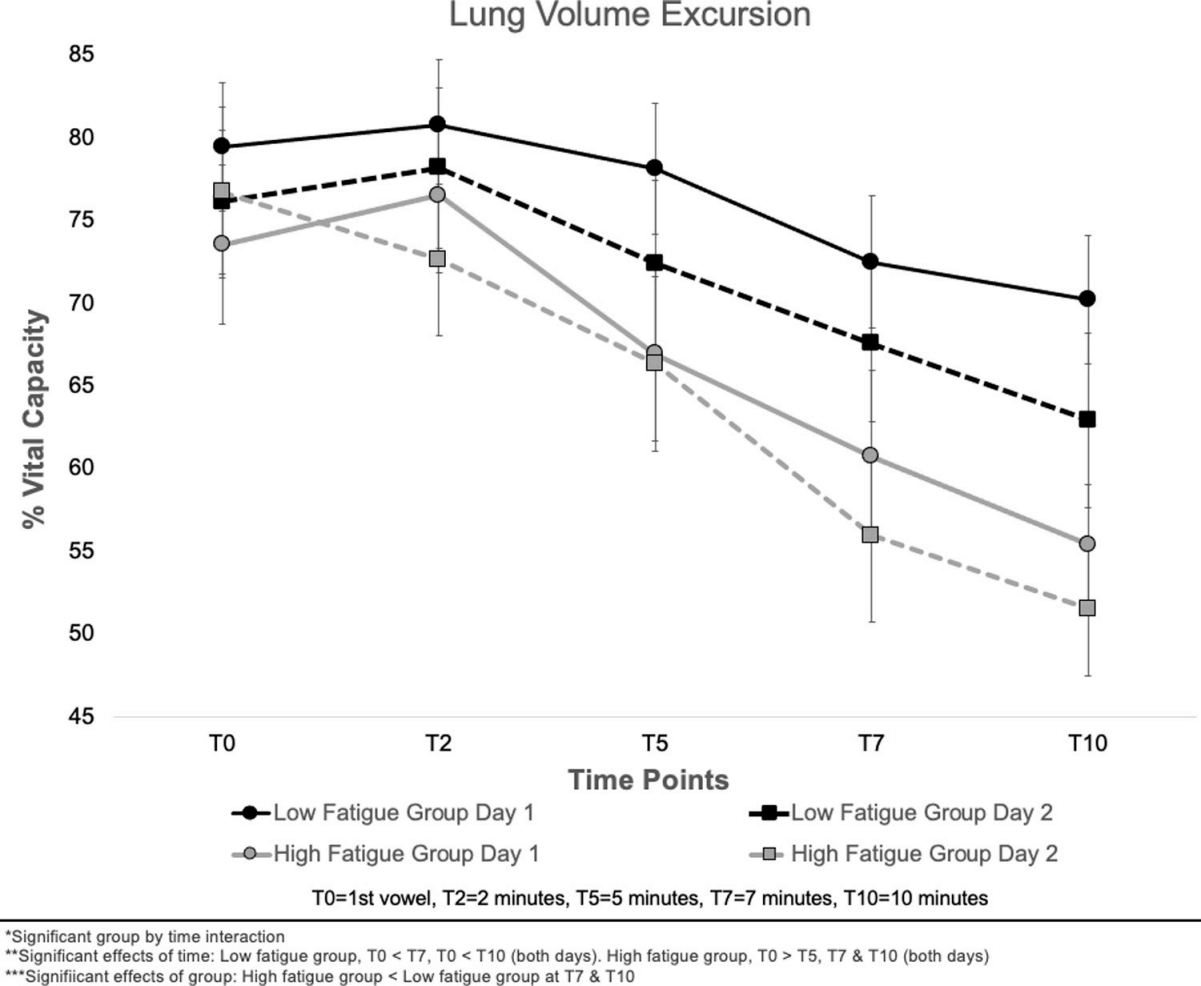
Means and standard errors for lung volume excursion across all time points, experimental days, and low and high vocal fatigue groups.

Means and standard errors for LVE are presented in Fig 5. A significant group by time interaction was observed for LVE (F (4, 112) = 2.68, *p* = .035). For the low vocal fatigue group, LVE was significantly lower at T7 (*p* = .004) and T10 (*p* < .001) as compared to T0 on both days. For the high vocal fatigue group, LVE decreased more quickly. As compared to T0, LVE was significantly lower at T5 (*p* = .038), T7 (*p* = .005), and T10 (*p* = .535). LVE data did not differ between the low and high vocal fatigue groups at T0 (*p* = .633), T2 (*p* = .717), or T5 (*p* = .193). LVE was significantly smaller for the high vocal fatigue group at T7 (*p* = .033) and T10 (*p* = .043). No significant main effects of group, day, or significant interaction of time, group, and day were observed.

### Acoustic measures

Means and standard errors for all acoustic outcome measures are reported in Table 1.

**Table 1:** Means and standard errors for acoustic outcome measures.

	Low Vocal Fatigue Group					High Vocal Fatigue Group				
	Time Points for Day 1^a^									
Measure	**T0**	**T2**	**T5**	**T7**	**T10**	**T0**	**T2**	**T5**	**T7**	**T10**
**Duration (sec)**	17.7 (1.1)	18.3 (1.6)	16.4 (1.5	15.9 (1.4)	14.5 (.81)	12.3 (1.1)	12.8 (1.0)	10.6 (.82)	11.0 (.77)	9.4 (.70)
**SPL (dB)** ^d^	89.8 (.92)	89.3 (1.0)	88.2 (1.3)	88.6 (1.2)	88.4 (1.2)	84.5 (1.2)	87.7 (.62)	86.5 (.65)	86.2 (1.0)	86.5 (.76)
**F0 (Hz)** ^c^	287.9 (15.0)	291.9 (14.1)	291.5 (13.8)	295.9 (14.1)	295.9 (14.4)	257.3 (22.9)	265.9 (23.1)	264.8 (25.3)	258.2 (25.0)	266.3 (26.8)
**CPP (dB)** ^b^	10.9 (.24)	11.4 (.26)	11.4 (.25)	11.3 (.24)	11.4 (.22)	8.8 (.38)	9.6 (.35)	9.3 (.38)	9.8 (.32)	9.5 (.31)
	Time Points for Day 2^a^									
Measure	**T0**	**T2**	**T5**	**T7**	**T10**	**T0**	**T2**	**T5**	**T7**	**T10**
**Duration (sec)**	19.7 (1.1)	15.1 (1.3)	15.7 (1.4)	13.3 (1.4)	13.0 (1.0)	12.5 (.75)	10.2 (1.2)	9.5 (.70)	8.9 (.51)	8.3 (.39)
**SPL (dB)** ^d^	86.1 (.84)	85.1 (.77)	84.2 (.76)	83.2 (.87)	82.2 (.85)	83.5 (.60)	84.4 (.84)	82.0 (.73)	80.6 (.67)	78.2 (.61)
**F0 (Hz)** ^c^	290.6 (14.7)	295.0 (14.3)	287.2 (15.4)	297.1 (13.3)	299.2 (14.2)	269.6 (19.5)	268.2 (20.6)	267.4 (20.9)	266.9 (21.1)	269.5 (22.0)
**CPP (dB)** ^b^	10.1 (.24)	10.6 (.26)	10.6 (.25)	10.5 (.24)	10.6 (.22)	8.5 (.38)	9.5 (.35)	9.1 (.38)	9.4 (.27)	9.2 (.29)

^a^T0 = first vowel of vocal exertion task, T2 = 2 minutes, T5 = 5 minutes, T7 = 7 minutes, T10 = 10 minutes

^b^CPP = cepstral peak prominence

^c^F0 = fundamental frequency

^d^SPL = sound pressure level

#### Duration of sustained vowel

A significant main effect of time was observed for duration of sustained vowel (F (4, 112) = 10.50, *p* < .0001). For both groups, vowel duration significantly decreased between T0 and T5 (*p* = .002). Values remained significantly below T0 at T7 (*p* < .001) and T10 (*p* < .001). A significant main effect of group was also observed for duration of sustained vowel (F (1, 28) = 13.72, *p* = .0009), as the low vocal fatigue group sustained significantly longer vowels at all time points (*p* < .001). A significant main effect of day was also observed for vowel duration (F (1, 28) = 8.85, *p* = .006). For both groups, no differences were observed between experimental days for vowel duration at T0 (low fatigue group *p* = .223; high fatigue group *p* = .101) however, on day two, vowels became significantly shorter by T2 (low fatigue group *p* < .01; high fatigue group *p* < .01). Data did not differ between experimental days at T5, but for day two durations were also significantly shorter at T7 (low fatigue group *p* < .01; high fatigue group *p* < .01) and T10 (low fatigue group *p* < .01; high fatigue group *p* < .01). No significant interactions were observed for vowel duration.

#### Sound pressure level (SPL)

A significant interaction between group and day was observed for SPL (F (1, 28) = 5.44, *p* = .0271). On day one, the low vocal fatigue group produced vowels at significantly higher SPL than the high fatigue group all time points (p < .001). On day two SPL was in similar range for both groups at T2 (*p* = .325) only and was greater for the low fatigue group than the high fatigue group at all other time points (*p* < .001). SPL was greater on day one when compared with day 2 for both groups at all time points (*p* < .01), except T0 for the high vocal fatigue group where SPL was similar on both days (*p* = .348). No significant main effect of time or interaction between day, time, and group were observed.

#### Fundamental frequency (F0)

No significant interactions or main effects were observed for F0.

#### Cepstral peak prominence (CPP)

**Fig 6:**
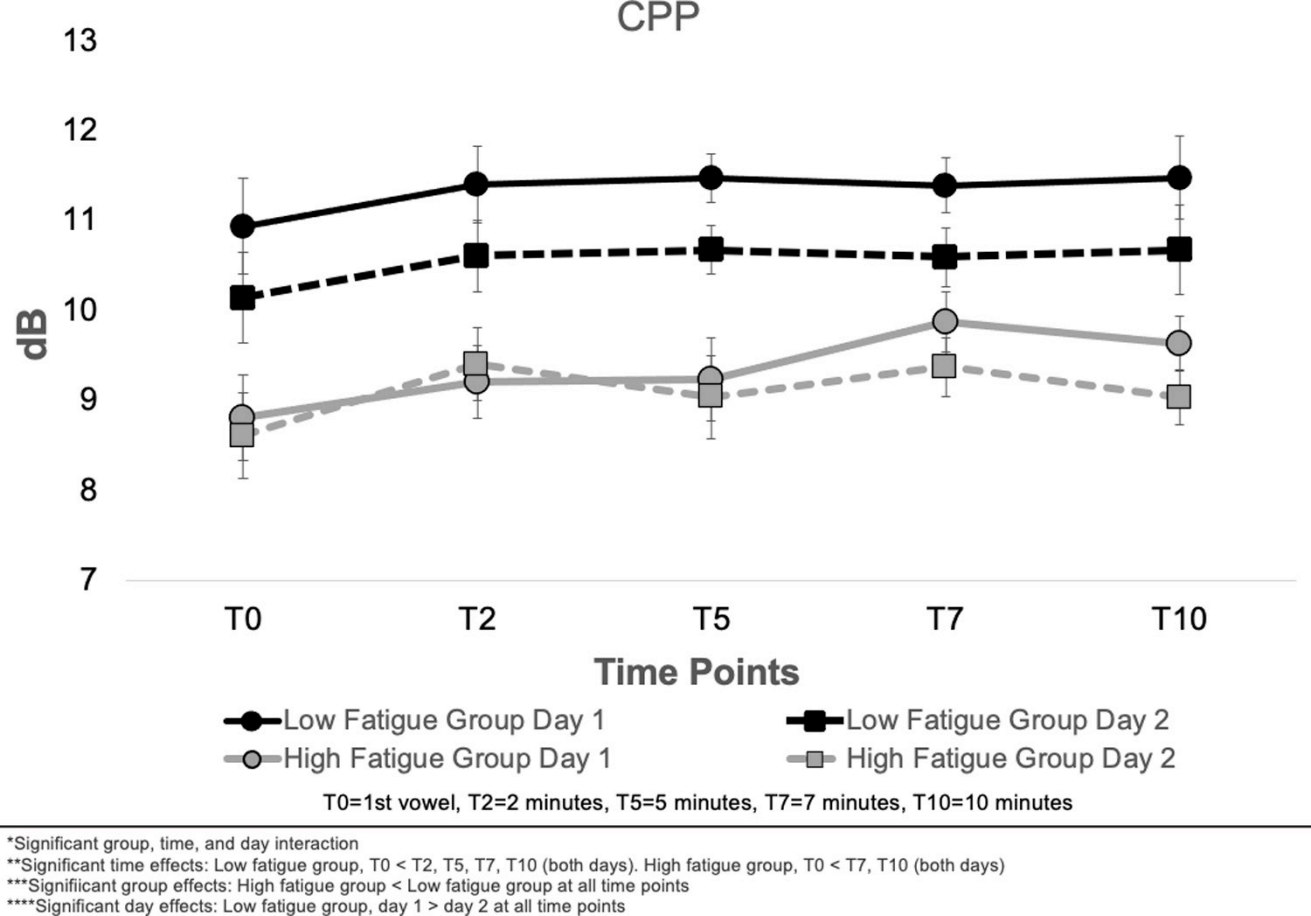
Means and standard errors for Cepstral Peak Prominence (CPP) across all time points, experimental days, and low and high vocal fatigue groups.

Means and standard errors for CPP are presented in Fig 6. A statistically significant three-way interaction between group, time, and day was observed for CPP (F (4, 112) = 2.75, *p* = .0317). CPP data were significantly greater for the low vocal fatigue group when compared with the high fatigue group at all time points (*p* < .01). For the low fatigue group, CPP was greater day one than day two at all time points (*p* < .001). On both days, CPP for the low vocal fatigue group became significantly greater than T0 at T2 (day one *p* = .036; day two *p* = .037) and remained significantly above T0 at all time points through T10 (day one *p* = .026; day two *p* = .037). For the high vocal fatigue group, no statistically significant differences were observed between days one and two. On both days, CPP at T7 was significantly greater than T0 (day one *p* = .035, day two *p* = .039) and remained significantly greater than T0 at T10 (day one *p* = .028; *p* = .008).

## Discussion

This study examined the respiratory strategies employed by individuals reporting low and high levels of vocal fatigue during an exertion task across two experimental days. It was hypothesized that individuals reporting high levels of vocal fatigue in daily life would utilize lower lung volumes to complete the vocal exertion task when compared to speakers reporting low levels of vocal fatigue. It was also hypothesized that these strategies might change when the vocal exertion task was performed a second time. No differences in respiratory kinematics were observed between groups at T0. As the exertion task progressed, individuals reporting high levels of vocal fatigue initiated phonation at lower lung volumes, while those with low levels of vocal fatigue initiated phonation at higher lung volumes. Overall lung volume excursion decreased as the task progressed (T2-T10) for both groups. Participants employed similar respiratory strategies on both experimental days.

It was also hypothesized that acoustic measures would be affected by the vocal exertion task. Differences in acoustic measures were observed between low and high vocal fatigue groups, both at T0 and as the exertion task progressed (T2-T10). Individuals reporting vocal fatigue symptoms produced shorter vowels at lower SPL than the low fatigue group on both experimental days. Vowels became shorter and softer on the second experimental day for both groups. Changes in cepstral peak prominence followed SPL for the low vocal fatigue group but did not change between experimental days for the high vocal fatigue group.

Seven minutes into the task (T7), individuals in the low vocal fatigue group began initiating phonation at higher lung volumes. This increase in LVI was likely a strategy employed to maintain elevated vocal intensity in the face of continued vocal loading. Increasing the lung volume used to initiate speech would allow individuals to benefit from greater recoil forces [24, 31, 62] and thereby potentially avoid vocal strain [17]. As previously published, these participants employed the same strategy following the task [17, 34], but the findings from this analysis suggest that this pattern of higher LVI is initiated during the actual vocal demand itself. In other words, the strategy was evident in the moment of vocal demand as well as following the demanding task. It might be predicted that compensations employed during vocal exertion would allow individuals to benefit from recoil forces and avoid compensatory strain. In fact, avoiding compensatory strain is at least a secondary target of most voice therapy regimens [63–67]. In addition, avoiding compensatory strain could be beneficial over an extended period of time, whereas vocal strain might quickly lead to negative vocal symptoms [68, 69]. This finding could, in part, be related to the type of vocal exertion task used in this study. Although evidence suggests that sustained vowel tasks fatigue the laryngeal mechanism more quickly than traditional loud reading tasks [17, 22], some of the effects may arise from the fact that the tasks differ from habitual phonatory patterns. Additional investigation is warranted to determine if speakers employ similar respiratory strategies for other types of vocal exertion.

For individuals reporting high vocal fatigue, a decrease in LVI was observed. This decrease in LVI would force individuals to produce loud, prolonged phonation without the benefit of increased recoil forces [24, 31, 62]. Individuals might then compensate on a laryngeal level with glottal squeeze or excess muscle tension [30, 34]. Such compensations could easily lead to vocal fatigue that becomes more severe over extended periods of high vocal demand. This hypothesis is supported by previous work reporting that phonating at low lung volumes is associated with functional voice problems [3, 70]. For example, teachers with vocal fatigue have been observed to use lower LVI for habitual teaching tasks [3]. As such, increased respiratory efficiency and unloading excess muscle recruitment during vocalization might be viable therapeutic targets [71, 72].

LVE decreased for both groups as vocal exertion task progressed. As a result, vowel duration decreased for both the low and high vocal fatigue groups five minutes (at T5) into the vocal exertion task. This might be expected as sustaining vowels for maximal phonation time is a physiologically-challenging task requiring extended airflow regulation, laryngeal valving, and respiratory muscle strength [73, 74]. The fact that participants sustained vowels for five minutes without decreasing vowel duration is likely due in part to the frequent cues provided during data collection, as past study has observed maximum phonation time to decrease in as few as five trials [75]. Although it is not possible to determine if the reason for the decrease in vowel duration was the result of physical fatigue, mental fatigue, or a combination of the two–participants subjectively reported increased vocal effort after the vocal exertion task [17, 34].

Interestingly, when tasked with sustaining prolonged vowels at elevated SPL and F0, participants maintained accurate SPL and F0 targets throughout the task on day one. It was, therefore, vowel duration that was compromised when all targets could no longer be achieved. This reflects an interesting allocation of resources in the setting of high vocal demand. This also explains why CPP remained constant over the course of the task, as both F0 and SPL have been shown to influence cepstral measures [76–78]. The change in vowel duration, rather than F0 or SPL, may have implications for how individuals compensate when required to communicate for extended periods in suboptimal environment. That is: they may breathe more frequently to maintain these other parameters. This would make sense as this pattern is also observed in loud connected speech or when speaking in background noise [31]. This warrants further investigation as poor acoustics or ambient noise can alter vocal effort and acoustic measures [79–81].

The high and low vocal fatigue groups displayed differences in acoustic measures at T0. This was unexpected as none of the participants were experiencing vocal symptoms at the time of the study. Nonetheless, individuals reporting high levels of vocal fatigue produced shorter vowels at T0 and throughout the exertion task despite receiving identical instructions and cues. It is unclear why this difference in loud maximum phonation time was evident between groups, particularly as lung volumes were similar between groups at T0. It may be that individuals experiencing frequent vocal symptoms, were less inclined to push themselves vocally for fear of experiencing vocal fatigue. Individuals reporting low amounts of vocal fatigue may have been less inhibited as they did not anticipate vocal problems. It is also possible that an underlying physiological or anatomical difference existed between these populations. Determining whether these differences result from or cause vocal fatigue should be the subject of future study. The fact that the lung volumes used to complete the task were similar at T0 suggests that the high vocal fatigue group produced shorter vowels with the same amount of air employed by the low fatigue group. Additional research may explain this finding and determine the potential screening value of loud maximum phonation time.

SPL was lower for the high vocal fatigue group at T0 but rose to near that of the low fatigue group 2 minutes (T2) into the vocal exertion task. This suggests that the high vocal fatigue group experienced a warm-up period before they could consistently sustain the vowel at target SPL but then SPL decreased again by the 5-minute marker (T5). CPP was significantly lower for the high vocal fatigue group at all time points. This finding was likely driven primarily by SPL rather than other factors known to influence CPP, such as sex-related differences. This conclusion is reasonable as the high vocal fatigue group was 60% male, which would normally increase CPP values as males have larger normative values on this measure than females [76].

SPL was the primary acoustic difference observed between experimental days, as both overall groups produced vowels at lower SPL on day two. The reasons for this difference are unclear given that participants were given the same cues and targets on both experimental days. It may be that participants were vocally pacing themselves after having experienced the vocal exertion task on the day one. The decrease in SPL on day two led to a decrease in CPP for the low vocal fatigue group. This was expected as vocal intensity is correlated with CPP values [76]. For the high vocal fatigue group, the expected decrease in CPP was not evident on day two. This may be because vocal quality became more pressed for the high vocal fatigue group at these time points. Past study indicates that phonating at lower lung volumes is associated with higher laryngeal position, lower tracheal pull, and therefore increased vocal fold adduction [82]–which could lead to slightly more pressed phonation [83–85]. Previous studies have shown that changes in voice quality can alter CPP [86–88], thus more pressed vocal quality may have counteracted the effects of decreased SPL. More work is needed to examine this physiological relationship between lower and upper airway. It should be noted that although values have been suggested for separating normal and dysphonic voices [88, 89], it is unclear what constitutes a clinically meaningful difference in CPP for healthy voices. It is possible that some of the smaller magnitude differences observed in this study are not clinically significant.

Given the decrease in SPL on day two, it is surprising that there were no significant differences in lung volumes between experimental days. However, SPL is also regulated by supraglottal adjustments which were not examined in this study [90]. It may be that increased vocal effort on the second experimental day caused individuals to use greater respiratory drive, as emerging evidence suggests that increased vocal effort can affect the lung volumes used for speech and glottal adduction [28]. Additional research is needed to understand why individuals used generally the same respiratory drive to produce lower SPL vowels on day two.

Data from this study suggest that individuals reporting high levels of vocal fatigue utilize less efficient respiratory strategies when faced with vocal challenge. This could make them more vulnerable to problematic compensations such as vocal strain. More work, however, will be needed to address some of the limitations of this study. For example, additional work will be needed to determine if the differences between low and high vocal fatigue groups are indicative of some underlying physiology or functional patterns developed over time. Ascertaining whether these patterns are causative of -or result from- vocal fatigue also warrants investigation. More work is also needed to determine the long-term implications of these respiratory strategies and their effects on laryngeal function. Specifically, future study incorporating electroglottographic measures may clarify the relationship between lung volumes and glottal closure. In addition, it will be important to examine more vulnerable populations such as older adults, those at occupational risk, or those with laryngeal pathology. This type of investigation may lead to better treatments for vocal fatigue.

## Conclusions

Individuals experiencing frequent vocal fatigue symptoms employed less efficient respiratory strategies during periods of high vocal demand when compared with individuals reporting low vocal fatigue. These findings suggest that respiratory kinematic changes previously observed following vocal exertion occur during periods of high vocal demand as well. In addition, individuals reporting high vocal fatigue produced softer, shorter vowels than those in the low vocal fatigue group, at baseline. Future research is warranted to consider the implications of these differences in vocal fatigue management.

## Supporting information

S1 Fig Schematic of the hypothesized relationship between vocal exertion, vocal fatigue, laryngeal function and respiratory kinematics. (TIF)

S1 File Study data. (DOCX)
