# Supplementary figures and images for: The effects of vocal exertion on lung volume measurements and acoustics in speakers reporting high and low vocal fatigue
Source: PLoS One. 2022 May 12;17(5):e0268324. doi: 10.1371/journal.pone.0268324 (PMC9098027; doi:10.1371/journal.pone.0268324)

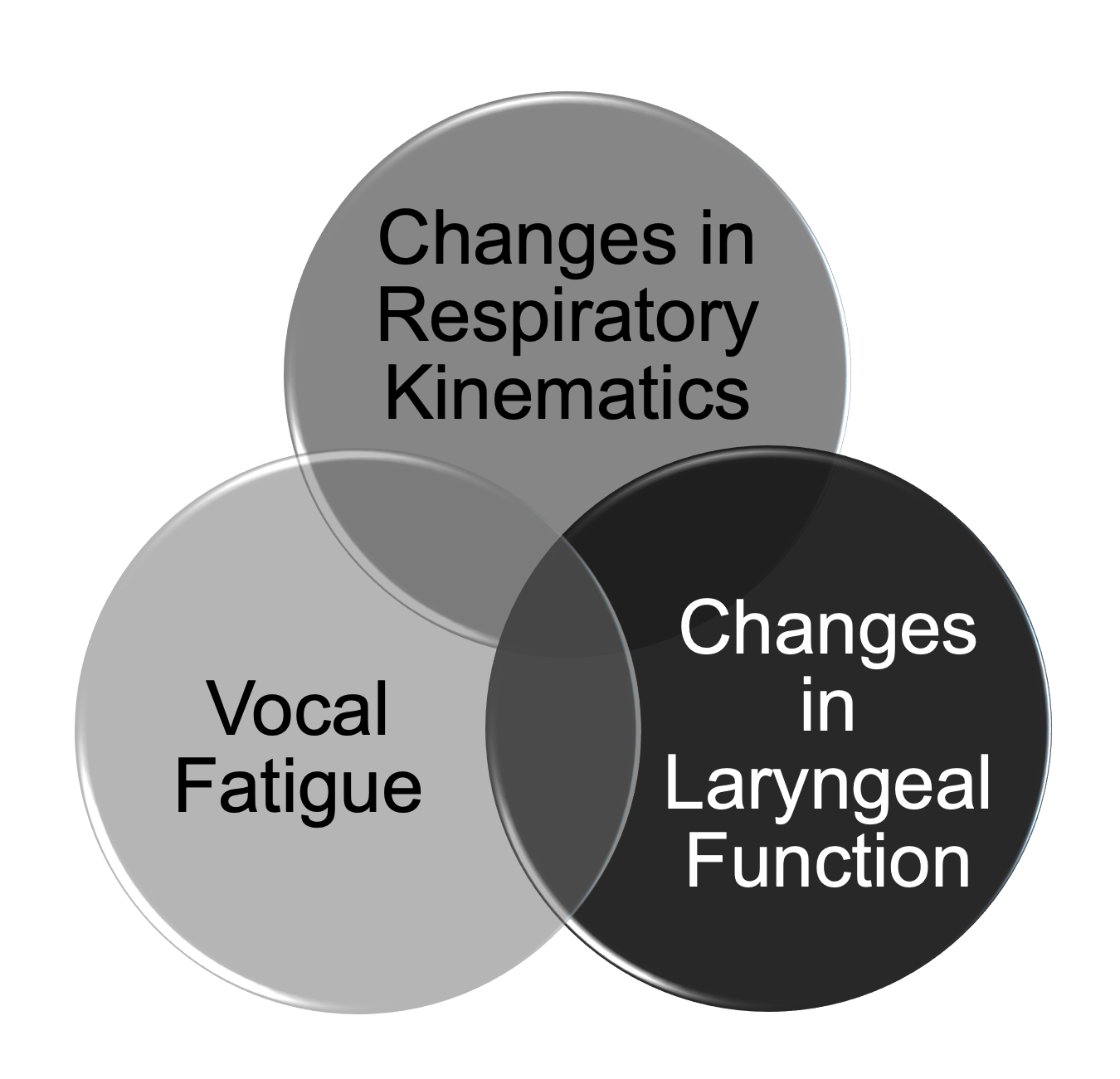

Supplement: S1 Fig — (TIF) [file pone.0268324.s001.tif]
