# Supplementary material for: The effects of vocal exertion on lung volume measurements and acoustics in speakers reporting high and low vocal fatigue
Source: PLoS One. 2022 May 12;17(5):e0268324. doi: 10.1371/journal.pone.0268324 (PMC9098027; doi:10.1371/journal.pone.0268324)
Supplement: S1 File — (DOCX) [file pone.0268324.s002.docx]

| **Low Fatigue** | | | Subject | | 1 | 2 | | 3 | | 4 | | 5 | 6 | | 7 | | 8 | | 9 | 10 | | 11 | | 12 | | 13 | | 14 | 15 | 16 | 17 | 18 | 19 | 20 |
| --- | --- | --- | --- | --- | --- | --- | --- | --- | --- | --- | --- | --- | --- | --- | --- | --- | --- | --- | --- | --- | --- | --- | --- | --- | --- | --- | --- | --- | --- | --- | --- | --- | --- | --- |
| Measure | Time Point | | | |  |  | |  | |  | |  |  | |  | |  | |  |  | |  | |  | |  | |  |  |  |  |  |  |  |
| CPP (dB) | Day 1 | | 1 | | 12.09 | 11.45 | | 11.37 | | 11.41 | | 7.85 | 9.95 | | 10.07 | | 10.62 | | 11.52 | 11.10 | | 11.85 | | 11.37 | | 11.39 | | 13.27 | 12.46 | 9.32 | 10.24 | 9.95 | 10.47 | 10.54 |
|  |  |  | 2 | | 12.23 | 10.73 | | 11.83 | | 11.97 | | 8.54 | 10.64 | | 10.76 | | 11.51 | | 12.96 | 11.60 | | 12.79 | | 11.80 | | 12.83 | | 13.77 | 12.79 | 11.48 | 9.08 | 10.15 | 9.10 | 11.41 |
|  |  |  | 5 | | 12.09 | 10.59 | | 12.52 | | 12.67 | | 9.49 | 11.59 | | 10.42 | | 10.82 | | 12.19 | 12.06 | | 12.85 | | 12.50 | | 12.06 | | 11.30 | 12.85 | 12.02 | 9.15 | 10.27 | 9.17 | 12.59 |
|  |  |  | 7 | | 12.06 | 10.56 | | 11.83 | | 12.25 | | 8.32 | 10.42 | | 10.42 | | 11.20 | | 12.58 | 11.67 | | 12.59 | | 12.36 | | 12.45 | | 12.19 | 12.59 | 11.76 | 10.48 | 9.12 | 10.50 | 11.83 |
|  |  |  | 10 | | 12.09 | 10.59 | | 12.29 | | 12.95 | | 9.32 | 11.42 | | 11.54 | | 10.22 | | 12.03 | 11.40 | | 12.49 | | 12.78 | | 11.90 | | 10.46 | 12.49 | 12.10 | 10.73 | 9.77 | 10.75 | 11.38 |
|  | Day 2 | | 1 | | 11.29 | 10.65 | | 10.57 | | 10.61 | | 7.05 | 9.15 | | 9.27 | | 9.82 | | 10.72 | 10.30 | | 11.05 | | 10.57 | | 10.59 | | 12.47 | 11.66 | 8.52 | 9.44 | 9.15 | 9.67 | 9.74 |
|  |  |  | 2 | | 11.43 | 9.93 | | 11.03 | | 11.17 | | 7.74 | 9.84 | | 9.96 | | 10.71 | | 12.16 | 10.80 | | 11.99 | | 11.00 | | 12.03 | | 12.97 | 11.99 | 10.68 | 8.28 | 9.35 | 8.30 | 10.61 |
|  |  |  | 5 | | 11.29 | 9.79 | | 11.72 | | 11.87 | | 8.69 | 10.79 | | 9.62 | | 10.02 | | 11.39 | 11.26 | | 12.05 | | 11.70 | | 11.26 | | 10.50 | 12.05 | 11.22 | 8.35 | 9.47 | 8.37 | 11.79 |
|  |  |  | 7 | | 11.26 | 9.76 | | 11.03 | | 11.45 | | 7.52 | 9.62 | | 9.62 | | 10.40 | | 11.78 | 10.87 | | 11.79 | | 11.56 | | 11.65 | | 11.39 | 11.79 | 10.96 | 9.68 | 8.32 | 9.70 | 11.03 |
|  |  |  | 10 | | 11.29 | 9.79 | | 11.49 | | 12.15 | | 8.52 | 10.62 | | 10.74 | | 9.42 | | 11.23 | 10.60 | | 11.69 | | 11.98 | | 11.10 | | 9.66 | 11.69 | 11.30 | 9.93 | 8.97 | 9.95 | 10.58 |
| Duration (seconds) | Day 1 | | 1 | | 21.50 | 21.36 | | 12.73 | | 23.06 | | 15.70 | 14.70 | | 30.56 | | 24.61 | | 12.11 | 20.28 | | 17.04 | | 25.55 | | 17.57 | | 16.23 | 17.89 | 14.82 | 12.34 | 11.78 | 12.33 | 12.15 |
|  |  |  | 2 | | 22.57 | 10.25 | | 10.70 | | 27.00 | | 13.77 | 8.86 | | 15.85 | | 20.45 | | 15.47 | 16.48 | | 17.93 | | 30.13 | | 16.10 | | 16.87 | 38.66 | 23.70 | 13.69 | 22.94 | 13.18 | 13.05 |
|  |  |  | 5 | | 18.88 | 10.80 | | 10.50 | | 14.06 | | 15.54 | 11.39 | | 13.36 | | 12.17 | | 14.67 | 13.79 | | 15.38 | | 26.00 | | 11.17 | | 18.37 | 38.46 | 24.42 | 10.96 | 25.25 | 11.99 | 11.40 |
|  |  |  | 7 | | 16.72 | 5.44 | | 10.90 | | 16.28 | | 13.44 | 12.06 | | 10.86 | | 15.46 | | 10.69 | 11.08 | | 11.74 | | 17.80 | | 14.53 | | 15.04 | 24.68 | 23.37 | 15.26 | 34.71 | 26.39 | 12.84 |
|  |  |  | 10 | | 13.98 | 9.32 | | 11.60 | | 14.77 | | 12.40 | 13.12 | | 10.76 | | 15.56 | | 11.02 | 15.02 | | 11.99 | | 22.21 | | 12.96 | | 13.55 | 15.06 | 16.95 | 15.25 | 25.39 | 15.61 | 15.04 |
|  | Day 2 | | 1 | | 21.24 | 21.77 | | 19.98 | | 20.81 | | 20.60 | 8.26 | | 16.25 | | 20.93 | | 18.19 | 20.63 | | 15.28 | | 21.77 | | 20.57 | | 12.99 | 27.76 | 21.17 | 13.08 | 30.20 | 25.81 | 17.76 |
|  |  |  | 2 | | 21.45 | 10.28 | | 9.93 | | 11.51 | | 18.80 | 8.89 | | 8.88 | | 12.99 | | 11.23 | 10.37 | | 12.62 | | 17.36 | | 21.26 | | 10.98 | 23.93 | 17.36 | 11.63 | 30.09 | 24.58 | 9.51 |
|  |  |  | 5 | | 26.75 | 10.48 | | 12.85 | | 18.38 | | 7.91 | 8.46 | | 8.92 | | 11.92 | | 16.12 | 12.68 | | 11.76 | | 14.36 | | 20.67 | | 11.42 | 25.73 | 23.13 | 11.67 | 29.79 | 21.51 | 10.32 |
|  |  |  | 7 | | 16.77 | 7.51 | | 12.56 | | 9.27 | | 6.80 | 7.76 | | 9.13 | | 9.80 | | 12.17 | 11.06 | | 11.92 | | 16.05 | | 16.48 | | 8.99 | 23.41 | 15.50 | 11.46 | 34.83 | 14.15 | 10.68 |
|  |  |  | 10 | | 19.32 | 8.59 | | 19.93 | | 18.70 | | 8.45 | 7.73 | | 8.75 | | 9.40 | | 9.65 | 10.33 | | 9.46 | | 16.77 | | 18.11 | | 9.28 | 16.13 | 19.50 | 11.72 | 18.48 | 11.38 | 9.80 |
| SPL (dB) | Day 1 | | 1 | | 86.00 | 85.04 | | 90.97 | | 85.83 | | 86.93 | 89.23 | | 92.42 | | 90.17 | | 92.30 | 91.96 | | 82.73 | | 93.53 | | 89.43 | | 83.25 | 89.06 | 97.77 | 94.91 | 93.65 | 85.30 | 96.36 |
|  |  |  | 2 | | 87.22 | 81.84 | | 93.82 | | 83.60 | | 89.82 | 91.38 | | 95.29 | | 90.31 | | 89.75 | 88.39 | | 84.31 | | 90.37 | | 88.77 | | 81.86 | 87.28 | 97.35 | 93.30 | 98.24 | 89.32 | 85.06 |
|  |  |  | 5 | | 85.83 | 81.43 | | 95.31 | | 83.80 | | 90.79 | 91.64 | | 88.06 | | 88.35 | | 88.62 | 82.85 | | 83.24 | | 93.51 | | 90.10 | | 78.17 | 87.52 | 102.13 | 91.22 | 98.46 | 84.08 | 80.01 |
|  |  |  | 7 | | 86.98 | 80.69 | | 94.94 | | 85.30 | | 92.08 | 89.36 | | 88.78 | | 91.45 | | 90.61 | 79.55 | | 86.29 | | 93.26 | | 92.40 | | 77.28 | 90.01 | 98.94 | 91.87 | 97.36 | 85.24 | 80.30 |
|  |  |  | 10 | | 85.01 | 81.70 | | 98.39 | | 84.92 | | 92.90 | 89.93 | | 88.48 | | 89.24 | | 87.49 | 83.17 | | 85.60 | | 91.57 | | 95.75 | | 79.98 | 86.77 | 97.06 | 91.38 | 95.73 | 83.14 | 80.25 |
|  | Day 2 | | 1 | | 86.00 | 85.04 | | 98.13 | | 88.01 | | 84.32 | 82.59 | | 88.94 | | 87.79 | | 86.89 | 82.43 | | 88.18 | | 89.96 | | 87.63 | | 84.43 | 81.10 | 80.87 | 83.16 | 85.43 | 85.00 | 87.83 |
|  |  |  | 2 | | 87.22 | 81.84 | | 93.82 | | 81.29 | | 88.81 | 83.65 | | 87.29 | | 86.58 | | 76.36 | 84.48 | | 83.19 | | 84.05 | | 86.65 | | 82.85 | 85.47 | 86.92 | 88.26 | 85.16 | 83.04 | 86.60 |
|  |  |  | 5 | | 85.83 | 81.43 | | 95.31 | | 84.52 | | 87.02 | 83.71 | | 84.38 | | 85.91 | | 82.10 | 82.76 | | 81.21 | | 84.20 | | 86.09 | | 82.85 | 84.19 | 80.50 | 79.92 | 86.36 | 79.61 | 86.80 |
|  |  |  | 7 | | 86.98 | 80.69 | | 94.94 | | 74.76 | | 84.66 | 80.31 | | 84.74 | | 84.49 | | 79.81 | 82.50 | | 80.99 | | 86.87 | | 83.82 | | 84.94 | 83.30 | 80.42 | 79.67 | 83.30 | 82.06 | 85.30 |
|  |  |  | 10 | | 85.01 | 81.70 | | 94.43 | | 78.65 | | 83.20 | 75.17 | | 81.59 | | 85.09 | | 78.64 | 82.23 | | 81.17 | | 76.84 | | 84.64 | | 83.17 | 82.66 | 82.51 | 80.46 | 81.72 | 80.75 | 84.92 |
| F0 (Hz) | Day 1 | | 1 | | 366.59 | 390.57 | | 340.88 | | 349.82 | | 326.37 | 265.27 | | 244.90 | | 264.48 | | 217.38 | 292.34 | | 343.86 | | 248.57 | | 135.14 | | 190.71 | 291.02 | 351.76 | 351.07 | 344.36 | 224.46 | 218.49 |
|  |  |  | 2 | | 366.22 | 384.24 | | 341.81 | | 348.20 | | 355.77 | 259.65 | | 259.97 | | 255.20 | | 217.44 | 289.98 | | 345.71 | | 246.83 | | 177.50 | | 196.37 | 281.87 | 361.12 | 350.84 | 347.38 | 226.01 | 226.63 |
|  |  |  | 5 | | 347.83 | 382.45 | | 352.84 | | 354.83 | | 351.63 | 252.12 | | 260.29 | | 252.30 | | 216.08 | 286.31 | | 347.57 | | 248.45 | | 196.35 | | 190.81 | 300.84 | 357.13 | 346.79 | 341.60 | 222.61 | 221.08 |
|  |  |  | 7 | | 355.73 | 387.84 | | 349.34 | | 346.19 | | 390.19 | 253.17 | | 259.82 | | 254.84 | | 223.22 | 285.27 | | 349.31 | | 245.68 | | 211.60 | | 182.34 | 317.60 | 364.40 | 343.22 | 338.08 | 236.56 | 223.59 |
|  |  |  | 10 | | 344.90 | 390.31 | | 362.58 | | 351.76 | | 388.05 | 259.33 | | 255.08 | | 239.91 | | 200.34 | 289.56 | | 349.43 | | 252.24 | | 223.89 | | 180.83 | 316.83 | 361.61 | 345.14 | 343.50 | 233.11 | 230.92 |
|  | Day 2 | | 1 | | 347.23 | 392.93 | | 354.31 | | 348.58 | | 326.13 | 260.07 | | 260.58 | | 267.45 | | 226.49 | 292.85 | | 352.13 | | 247.16 | | 139.62 | | 188.02 | 304.17 | 352.66 | 348.82 | 348.43 | 230.01 | 225.71 |
|  |  |  | 2 | | 352.28 | 384.64 | | 351.16 | | 337.31 | | 357.00 | 259.83 | | 262.34 | | 257.39 | | 222.27 | 291.55 | | 346.37 | | 249.85 | | 209.74 | | 155.54 | 329.27 | 375.49 | 350.52 | 345.95 | 231.03 | 231.64 |
|  |  |  | 5 | | 362.59 | 368.47 | | 354.48 | | 347.05 | | 356.54 | 253.78 | | 259.73 | | 259.97 | | 205.54 | 289.29 | | 174.32 | | 252.46 | | 206.48 | | 168.81 | 346.58 | 384.68 | 347.72 | 346.27 | 229.24 | 231.82 |
|  |  |  | 7 | | 362.91 | 386.95 | | 346.63 | | 346.84 | | 332.63 | 248.42 | | 259.01 | | 252.67 | | 216.50 | 289.03 | | 349.86 | | 246.90 | | 212.66 | | 200.80 | 358.78 | 354.05 | 347.37 | 347.76 | 241.51 | 241.30 |
|  |  |  | 10 | | 364.40 | 391.55 | | 349.55 | | 323.75 | | 327.64 | 254.86 | | 260.25 | | 249.40 | | 209.00 | 289.53 | | 351.51 | | 248.44 | | 209.99 | | 198.02 | 372.66 | 400.70 | 346.18 | 350.10 | 246.45 | 240.12 |
| LVI (%VC) | Day 1 | | 1 | | 38.93 | 48.55 | | 33.09 | | 36.00 | | 44.91 | 71.90 | | 61.78 | | 39.68 | | 34.71 | 42.82 | | 65.52 | | 58.61 | | 31.53 | | 49.60 | 40.15 | 54.56 | 63.63 | 50.62 | 47.07 | 29.52 |
|  |  |  | 2 | | 47.18 | 46.27 | | 24.26 | | 36.19 | | 37.95 | 60.46 | | 54.65 | | 34.40 | | 31.16 | 45.93 | | 76.29 | | 60.17 | | 29.67 | | 42.60 | 43.97 | 56.32 | 90.41 | 70.48 | 60.61 | 39.28 |
|  |  |  | 5 | | 52.13 | 75.65 | | 29.33 | | 37.84 | | 44.96 | 64.03 | | 58.74 | | 29.41 | | 36.26 | 38.28 | | 57.76 | | 65.29 | | 37.76 | | 27.30 | 41.68 | 54.11 | 106.11 | 41.68 | 42.85 | 48.54 |
|  |  |  | 7 | | 35.39 | 52.91 | | 27.44 | | 23.80 | | 74.37 | 83.96 | | 60.30 | | 20.88 | | 32.38 | 18.72 | | 102.00 | | 61.13 | | 13.73 | | 19.04 | 45.86 | 59.94 | 86.51 | 68.14 | 67.36 | 46.61 |
|  |  |  | 10 | | 28.63 | 50.78 | | 27.24 | | 22.03 | | 29.36 | 84.97 | | 66.04 | | 43.16 | | 66.93 | 26.17 | | 88.66 | | 61.50 | | 14.80 | | 19.28 | 45.68 | 70.31 | 113.99 | 54.20 | 66.72 | 48.67 |
|  | Day 2 | | 1 | | 38.93 | 48.55 | | 33.09 | | 26.00 | | 44.91 | 74.90 | | 61.78 | | 40.68 | | 34.71 | 42.82 | | 65.52 | | 58.61 | | 31.53 | | 49.60 | 20.15 | 54.56 | 63.63 | 34.62 | 38.07 | 29.52 |
|  |  |  | 2 | | 47.18 | 46.27 | | 24.26 | | 16.19 | | 37.95 | 60.46 | | 54.65 | | 24.40 | | 31.16 | 45.93 | | 76.29 | | 60.17 | | 29.67 | | 42.60 | 43.97 | 56.32 | 90.41 | 70.48 | 60.61 | 39.28 |
|  |  |  | 5 | | 52.13 | 75.65 | | 29.33 | | 27.84 | | 44.96 | 64.03 | | 58.74 | | 29.41 | | 26.26 | 38.28 | | 57.76 | | 65.29 | | 37.76 | | 27.30 | 41.68 | 54.11 | 106.11 | 41.68 | 42.85 | 48.54 |
|  |  |  | 7 | | 35.39 | 52.91 | | 27.44 | | 13.80 | | 54.37 | 83.96 | | 60.30 | | 20.88 | | 32.38 | 18.72 | | 102.00 | | 61.13 | | 13.73 | | 19.04 | 45.86 | 59.94 | 86.51 | 68.14 | 67.36 | 46.61 |
|  |  |  | 10 | | 28.63 | 50.78 | | 27.24 | | 12.03 | | 29.36 | 84.97 | | 66.04 | | 33.16 | | 66.93 | 26.17 | | 88.66 | | 61.50 | | 14.80 | | 19.28 | 45.68 | 60.31 | 99.99 | 54.20 | 66.72 | 48.67 |
| LVT (%VC) | Day 1 | | 1 | | 6.28 | -29.54 | | -7.21 | | -36.87 | | -47.67 | -56.73 | | -55.15 | | -79.43 | | -53.17 | -52.00 | | -20.26 | | -19.90 | | -63.63 | | -27.48 | -41.17 | -41.37 | -19.14 | -33.86 | -51.57 | -29.88 |
|  |  |  | 2 | | 21.87 | 12.68 | | -6.35 | | 1.58 | | -41.14 | -54.37 | | -57.90 | | -87.73 | | -44.07 | -51.20 | | -27.15 | | -23.08 | | -81.20 | | -37.03 | -42.45 | -43.83 | -5.44 | -15.45 | -45.13 | -21.23 |
|  |  |  | 5 | | 17.81 | 3.77 | | -6.60 | | 4.16 | | -51.30 | -39.83 | | -50.05 | | -81.33 | | -52.85 | -19.10 | | -9.73 | | -23.50 | | -79.03 | | -35.37 | -27.05 | -42.67 | 10.43 | -24.71 | -46.86 | -4.44 |
|  |  |  | 7 | | -1.59 | -5.90 | | -6.98 | | -8.76 | | -23.29 | -42.86 | | -49.77 | | -86.08 | | -43.54 | -39.45 | | 24.70 | | -35.94 | | -85.93 | | -41.42 | -21.12 | -11.83 | 18.84 | -29.66 | 0.97 | -14.34 |
|  |  |  | 10 | | -5.66 | 33.84 | | -6.24 | | -2.66 | | -19.52 | -21.07 | | -48.54 | | -51.02 | | -23.07 | -54.92 | | 17.59 | | -25.07 | | -88.93 | | -46.36 | -26.27 | -19.03 | 27.78 | -18.47 | 7.06 | -29.48 |
|  | Day 2 | | 1 | | 6.11 | -39.71 | | -7.38 | | -37.04 | | -47.84 | -56.90 | | -55.32 | | -79.60 | | -53.34 | -52.17 | | -20.43 | | -20.07 | | -63.80 | | -27.65 | -41.34 | -41.54 | -29.31 | -34.03 | -51.74 | -30.05 |
|  |  |  | 2 | | 21.70 | 12.51 | | -6.52 | | 1.41 | | -41.31 | -54.54 | | -58.07 | | -87.90 | | -44.24 | -51.37 | | -27.32 | | -23.25 | | -81.37 | | -37.20 | -42.62 | -44.00 | -5.61 | -15.62 | -45.30 | -21.40 |
|  |  |  | 5 | | 7.64 | 3.60 | | -6.77 | | 3.99 | | -51.47 | -40.00 | | -50.22 | | -81.50 | | -53.02 | -19.27 | | -9.90 | | -23.67 | | -79.20 | | -35.54 | -27.22 | -42.84 | 10.26 | -24.88 | -47.03 | -4.61 |
|  |  |  | 7 | | 1.42 | -6.07 | | -7.15 | | -8.93 | | -23.46 | -52.03 | | -49.94 | | -86.25 | | -43.71 | -39.62 | | 24.53 | | -36.11 | | -86.10 | | -41.59 | -21.29 | -12.00 | 22.67 | -29.83 | 0.80 | -15.51 |
|  |  |  | 10 | | -5.83 | 33.67 | | -6.41 | | -2.83 | | -19.69 | -21.24 | | -48.71 | | -51.19 | | -23.24 | -55.09 | | 17.42 | | -25.24 | | -89.10 | | -46.53 | -26.44 | -19.20 | 27.61 | -18.64 | 6.89 | -29.65 |
| **Vocal Fatigue** | | Subject | | 1 | | | 2 | | 3 | | 4 | | | 5 | | 6 | | 7 | | | 8 | | 9 | | 10 | |  |  |  |  |  |  |  |  |
| Measure | Time Point | | |  | | |  | |  | |  | | |  | |  | |  | | |  | |  | |  | |  |  |  |  |  |  |  |  |
| CPP (dB) | Day 1 | 1 | | 9.82 | | | 8.72 | | 10.77 | | 8.72 | | | 10.02 | | 6.57 | | 7.46 | | | 6.21 | | 10.42 | | 9.39 | |  |  |  |  |  |  |  |  |
|  |  | 2 | | 9.71 | | | 10.88 | | 11.54 | | 8.50 | | | 10.81 | | 7.91 | | 7.94 | | | 9.01 | | 10.19 | | 8.58 | |  |  |  |  |  |  |  |  |
|  |  | 5 | | 10.02 | | | 8.72 | | 11.03 | | 8.56 | | | 11.99 | | 7.35 | | 7.38 | | | 8.45 | | 10.25 | | 8.65 | |  |  |  |  |  |  |  |  |
|  |  | 7 | | 11.66 | | | 8.72 | | 10.82 | | 9.89 | | | 11.23 | | 8.55 | | 8.58 | | | 9.65 | | 9.69 | | 9.98 | |  |  |  |  |  |  |  |  |
|  |  | 10 | | 9.42 | | | 9.72 | | 10.98 | | 10.14 | | | 10.78 | | 8.02 | | 8.05 | | | 9.12 | | 9.89 | | 10.23 | |  |  |  |  |  |  |  |  |
|  | Day 2 | 1 | | 9.62 | | | 8.52 | | 10.57 | | 8.52 | | | 9.82 | | 6.37 | | 7.26 | | | 6.01 | | 10.22 | | 9.19 | |  |  |  |  |  |  |  |  |
|  |  | 2 | | 10.51 | | | 10.68 | | 11.34 | | 8.30 | | | 10.61 | | 7.71 | | 7.74 | | | 8.81 | | 9.99 | | 8.38 | |  |  |  |  |  |  |  |  |
|  |  | 5 | | 9.82 | | | 8.52 | | 10.83 | | 8.36 | | | 11.79 | | 7.15 | | 7.18 | | | 8.25 | | 10.05 | | 8.45 | |  |  |  |  |  |  |  |  |
|  |  | 7 | | 10.46 | | | 8.52 | | 10.62 | | 9.69 | | | 10.03 | | 8.35 | | 8.38 | | | 9.45 | | 9.49 | | 9.78 | |  |  |  |  |  |  |  |  |
|  |  | 10 | | 9.22 | | | 9.52 | | 10.78 | | 9.94 | | | 9.58 | | 7.82 | | 7.85 | | | 8.92 | | 9.69 | | 10.03 | |  |  |  |  |  |  |  |  |
| Duration (seconds) | Day 1 | 1 | | 13.56 | | | 9.09 | | 16.17 | | 10.35 | | | 11.04 | | 18.75 | | 17.22 | | | 8.40 | | 7.31 | | 11.74 | |  |  |  |  |  |  |  |  |
|  |  | 2 | | 10.21 | | | 15.99 | | 18.17 | | 8.64 | | | 13.08 | | 17.38 | | 15.70 | | | 9.38 | | 9.31 | | 11.02 | |  |  |  |  |  |  |  |  |
|  |  | 5 | | 11.00 | | | 14.57 | | 11.41 | | 9.19 | | | 13.63 | | 14.26 | | 9.03 | | | 6.65 | | 8.67 | | 8.36 | |  |  |  |  |  |  |  |  |
|  |  | 7 | | 7.69 | | | 14.68 | | 11.21 | | 11.72 | | | 12.79 | | 15.13 | | 9.54 | | | 8.54 | | 8.63 | | 10.08 | |  |  |  |  |  |  |  |  |
|  |  | 10 | | 7.37 | | | 9.94 | | 7.87 | | 8.94 | | | 6.51 | | 14.16 | | 7.60 | | | 11.35 | | 9.47 | | 11.47 | |  |  |  |  |  |  |  |  |
|  | Day 2 | 1 | | 14.81 | | | 16.67 | | 10.47 | | 9.06 | | | 10.91 | | 13.44 | | 11.75 | | | 12.99 | | 10.08 | | 15.40 | |  |  |  |  |  |  |  |  |
|  |  | 2 | | 19.84 | | | 12.60 | | 6.34 | | 7.18 | | | 13.22 | | 8.56 | | 7.98 | | | 7.93 | | 6.62 | | 12.62 | |  |  |  |  |  |  |  |  |
|  |  | 5 | | 15.35 | | | 9.91 | | 8.07 | | 8.52 | | | 10.96 | | 9.53 | | 7.41 | | | 9.38 | | 7.15 | | 9.49 | |  |  |  |  |  |  |  |  |
|  |  | 7 | | 12.44 | | | 9.53 | | 6.41 | | 8.85 | | | 9.99 | | 8.85 | | 6.84 | | | 7.72 | | 8.91 | | 9.87 | |  |  |  |  |  |  |  |  |
|  |  | 10 | | 9.92 | | | 9.62 | | 6.40 | | 8.68 | | | 7.69 | | 7.98 | | 9.33 | | | 6.91 | | 7.00 | | 9.87 | |  |  |  |  |  |  |  |  |
| SPL (dB) | Day 1 | 1 | | 80.64 | | | 82.09 | | 90.80 | | 83.72 | | | 90.92 | | 83.57 | | 82.93 | | | 82.69 | | 79.75 | | 88.44 | |  |  |  |  |  |  |  |  |
|  |  | 2 | | 84.57 | | | 90.59 | | 90.46 | | 87.03 | | | 90.35 | | 86.12 | | 87.93 | | | 86.38 | | 86.47 | | 87.85 | |  |  |  |  |  |  |  |  |
|  |  | 5 | | 87.42 | | | 85.46 | | 85.87 | | 88.31 | | | 89.14 | | 88.89 | | 86.50 | | | 83.95 | | 82.40 | | 87.56 | |  |  |  |  |  |  |  |  |
|  |  | 7 | | 87.01 | | | 93.13 | | 87.98 | | 83.01 | | | 89.40 | | 86.67 | | 85.62 | | | 83.41 | | 81.01 | | 85.14 | |  |  |  |  |  |  |  |  |
|  |  | 10 | | 88.47 | | | 91.22 | | 86.34 | | 85.39 | | | 88.25 | | 86.61 | | 84.89 | | | 86.45 | | 81.42 | | 85.96 | |  |  |  |  |  |  |  |  |
|  | Day 2 | 1 | | 79.84 | | | 85.84 | | 85.56 | | 81.10 | | | 84.32 | | 85.63 | | 84.29 | | | 82.97 | | 82.25 | | 83.41 | |  |  |  |  |  |  |  |  |
|  |  | 2 | | 80.52 | | | 86.52 | | 86.16 | | 84.46 | | | 87.14 | | 84.81 | | 82.42 | | | 80.45 | | 82.92 | | 88.78 | |  |  |  |  |  |  |  |  |
|  |  | 5 | | 86.01 | | | 82.37 | | 83.01 | | 81.10 | | | 86.36 | | 80.19 | | 81.73 | | | 79.14 | | 79.98 | | 80.78 | |  |  |  |  |  |  |  |  |
|  |  | 7 | | 83.87 | | | 79.87 | | 79.42 | | 80.14 | | | 85.56 | | 79.19 | | 80.35 | | | 79.47 | | 78.48 | | 79.72 | |  |  |  |  |  |  |  |  |
|  |  | 10 | | 82.01 | | | 77.01 | | 77.12 | | 77.16 | | | 76.26 | | 80.86 | | 78.37 | | | 79.27 | | 79.08 | | 75.69 | |  |  |  |  |  |  |  |  |
| F0 (Hz) | Day 1 | 1 | | 359.63 | | | 263.13 | | 132.86 | | 196.60 | | | 262.06 | | 324.12 | | 344.21 | | | 289.18 | | 242.87 | | 159.25 | |  |  |  |  |  |  |  |  |
|  |  | 2 | | 384.57 | | | 265.37 | | 149.15 | | 195.93 | | | 265.00 | | 329.39 | | 343.04 | | | 311.89 | | 239.70 | | 175.11 | |  |  |  |  |  |  |  |  |
|  |  | 5 | | 381.69 | | | 261.98 | | 122.86 | | 193.53 | | | 264.73 | | 326.67 | | 344.60 | | | 342.07 | | 238.26 | | 171.70 | |  |  |  |  |  |  |  |  |
|  |  | 7 | | 375.95 | | | 258.13 | | 112.41 | | 195.64 | | | 263.35 | | 344.18 | | 345.20 | | | 282.33 | | 234.96 | | 170.23 | |  |  |  |  |  |  |  |  |
|  |  | 10 | | 368.75 | | | 252.51 | | 111.06 | | 195.66 | | | 261.79 | | 344.90 | | 344.19 | | | 371.53 | | 238.56 | | 174.74 | |  |  |  |  |  |  |  |  |
|  | Day 2 | 1 | | 354.44 | | | 263.66 | | 202.97 | | 202.97 | | | 263.07 | | 327.26 | | 345.49 | | | 321.04 | | 243.39 | | 172.15 | |  |  |  |  |  |  |  |  |
|  |  | 2 | | 351.42 | | | 261.01 | | 199.10 | | 199.10 | | | 265.02 | | 330.38 | | 345.57 | | | 339.47 | | 216.51 | | 174.96 | |  |  |  |  |  |  |  |  |
|  |  | 5 | | 353.17 | | | 256.64 | | 197.56 | | 197.56 | | | 266.94 | | 325.89 | | 346.48 | | | 342.02 | | 212.75 | | 175.66 | |  |  |  |  |  |  |  |  |
|  |  | 7 | | 346.61 | | | 249.64 | | 196.70 | | 196.70 | | | 265.69 | | 333.52 | | 347.29 | | | 344.76 | | 214.43 | | 174.16 | |  |  |  |  |  |  |  |  |
|  |  | 10 | | 351.09 | | | 249.03 | | 198.50 | | 198.50 | | | 263.52 | | 337.73 | | 347.13 | | | 362.31 | | 212.19 | | 175.85 | |  |  |  |  |  |  |  |  |
| LVI (%VC) | Day 1 | 1 | | 37.81 | | | 55.31 | | 64.68 | | 62.64 | | | 63.34 | | 28.63 | | 39.98 | | | 44.96 | | 43.10 | | 44.07 | |  |  |  |  |  |  |  |  |
|  |  | 2 | | 32.74 | | | 37.39 | | 64.39 | | 56.42 | | | 56.80 | | 36.00 | | 54.18 | | | 39.65 | | 50.74 | | 42.95 | |  |  |  |  |  |  |  |  |
|  |  | 5 | | 32.96 | | | 31.03 | | 63.02 | | 48.42 | | | 68.16 | | 27.46 | | 43.37 | | | 57.19 | | 44.75 | | 39.85 | |  |  |  |  |  |  |  |  |
|  |  | 7 | | 34.69 | | | 37.71 | | 46.74 | | 52.23 | | | 55.99 | | 18.58 | | 38.18 | | | 43.03 | | 29.15 | | 43.09 | |  |  |  |  |  |  |  |  |
|  |  | 10 | | 19.98 | | | 34.27 | | 40.34 | | 33.70 | | | 30.54 | | 16.18 | | 42.44 | | | 38.86 | | 28.56 | | 39.66 | |  |  |  |  |  |  |  |  |
|  | Day 2 | 1 | | 33.46 | | | 55.31 | | 54.68 | | 62.64 | | | 63.34 | | 28.63 | | 39.98 | | | 44.96 | | 43.10 | | 44.07 | |  |  |  |  |  |  |  |  |
|  |  | 2 | | 32.96 | | | 37.39 | | 64.39 | | 56.42 | | | 56.80 | | 36.00 | | 34.18 | | | 39.65 | | 50.74 | | 42.95 | |  |  |  |  |  |  |  |  |
|  |  | 5 | | 34.69 | | | 31.03 | | 63.02 | | 48.42 | | | 68.16 | | 17.46 | | 43.37 | | | 57.19 | | 44.75 | | 39.85 | |  |  |  |  |  |  |  |  |
|  |  | 7 | | 32.74 | | | 37.71 | | 52.24 | | 32.23 | | | 65.99 | | 8.58 | | 38.18 | | | 43.03 | | 29.15 | | 33.09 | |  |  |  |  |  |  |  |  |
|  |  | 10 | | 19.98 | | | 34.27 | | 40.84 | | 33.70 | | | 40.54 | | 15.18 | | 32.44 | | | 38.86 | | 26.56 | | 25.66 | |  |  |  |  |  |  |  |  |
| LVT (%VC) | Day 1 | 1 | | -44.20 | | | 9.98 | | -40.55 | | -29.63 | | | -33.16 | | -36.70 | | -69.48 | | | -18.88 | | -35.05 | | -65.65 | |  |  |  |  |  |  |  |  |
|  |  | 2 | | -34.53 | | | -10.92 | | -19.71 | | -17.56 | | | -24.68 | | -49.09 | | -62.90 | | | -35.42 | | -31.97 | | -60.10 | |  |  |  |  |  |  |  |  |
|  |  | 5 | | -5.74 | | | -18.33 | | -9.57 | | -20.53 | | | 9.05 | | -65.46 | | -11.21 | | | -36.68 | | -34.25 | | -50.64 | |  |  |  |  |  |  |  |  |
|  |  | 7 | | -4.70 | | | -2.96 | | -1.90 | | -30.64 | | | 15.45 | | -94.99 | | 7.68 | | | -8.96 | | -29.70 | | -59.03 | |  |  |  |  |  |  |  |  |
|  |  | 10 | | 12.13 | | | -0.78 | | 6.60 | | -35.55 | | | 25.05 | | -16.31 | | -59.07 | | | -17.80 | | -32.61 | | -36.50 | |  |  |  |  |  |  |  |  |
|  | Day 2 | 1 | | -44.22 | | | 9.96 | | -40.57 | | -29.65 | | | -33.18 | | -36.72 | | -69.50 | | | -18.90 | | -35.07 | | -65.67 | |  |  |  |  |  |  |  |  |
|  |  | 2 | | -34.55 | | | -10.94 | | -19.73 | | -17.58 | | | -24.70 | | -49.11 | | -62.92 | | | -35.44 | | -31.99 | | -60.12 | |  |  |  |  |  |  |  |  |
|  |  | 5 | | -5.76 | | | -18.35 | | -9.59 | | -20.55 | | | 9.03 | | -65.48 | | -11.23 | | | -36.70 | | -34.27 | | -50.66 | |  |  |  |  |  |  |  |  |
|  |  | 7 | | -4.72 | | | -2.98 | | -1.92 | | -30.66 | | | 15.43 | | -95.01 | | 7.66 | | | -8.98 | | -29.72 | | -59.05 | |  |  |  |  |  |  |  |  |
|  |  | 10 | | 12.11 | | | -0.80 | | 6.58 | | -35.57 | | | 25.03 | | -16.33 | | -9.09 | | | -17.82 | | -32.63 | | -36.52 | |  |  |  |  |  |  |  |  |
